# Overview of Micro- and Nano-Technology Tools for Stem Cell Applications: Micropatterned and Microelectronic Devices

**DOI:** 10.3390/s121115947

**Published:** 2012-11-19

**Authors:** Stefano Cagnin, Elisa Cimetta, Carlotta Guiducci, Paolo Martini, Gerolamo Lanfranchi

**Affiliations:** 1 Department of Biology and CRIBI Biotechnology Centre, University of Padova, via U. Bassi 58/B, 35121 Padova, Italy; E-Mails: stefanoc@cribi.unipd.it (S.C.); paolom@cribi.unipd.it (P.M.); 2 Department of Biomedical Engineering, Columbia University, Vanderbilt Clinic Room 12-234, 630 West 168th Street, New York, NY 10032, USA; E-Mail: ec2438@columbia.edu; 3 CLSE IBI-EPFL, Swiss Up Chair on Engineering, Laboratory of Life Sciences Electronics, Institute of Bioengineering, Ecole polytechnique Fédérale de Lausanne BM 2106, Station 17, CH-1015 Lausanne, Switzerland; E-Mail: carlotta.guiducci@epfl.ch

**Keywords:** cell biosensor, micropattern, stem cell, cell microelectronic chip, cell microarray, microbioreactor

## Abstract

In the past few decades the scientific community has been recognizing the paramount role of the cell microenvironment in determining cell behavior. In parallel, the study of human stem cells for their potential therapeutic applications has been progressing constantly. The use of advanced technologies, enabling one to mimic the *in vivo* stem cell microenviroment and to study stem cell physiology and physio-pathology, in settings that better predict human cell biology, is becoming the object of much research effort. In this review we will detail the most relevant and recent advances in the field of biosensors and micro- and nano-technologies in general, highlighting advantages and disadvantages. Particular attention will be devoted to those applications employing stem cells as a sensing element.

## 1. Introduction

Research and advances in biosensor science experienced an exponential growth during the second half of the 20th century, boosted by their potential in detecting functional information from living cells. Advances in silicon micromachining, genomics, and cell culture technology have promoted research in the field of cell-based biosensors. From a mere technological standpoint, the adoption of soft lithographic techniques to fabricate cell chips brought advantages such as material compatibility with most biological assays, and ease of fabrication without the need to access advanced clean room facilities similar to those used for microelectronics. According to a recent on-line survey, the global biochips market is forecasted to reach US$9.1 billion by 2015 with a Compounded Annual Growth Rate (CAGR) of 20.9% during the 2009–2015 period [1]. The promise of this forecast is thus of paramount importance for companies producing instruments and consumables for biochips as well as for research organizations prevalently involved in miniaturization, drug discovery and biological systems in general. DNA microarrays revolutionized the analysis of gene expression and the identification of altered pathways in specific pathologies, making them currently used for patient categorization and clinical screening (reviewed in [2]). Their establishment derived from a decade-long research period during which scientists of different areas contributed to their optimal development, standardization [3] and the final conversion from specific research products into medical tools [2]. Similarly to DNA microarrays, cell-based chips may possibly replace classic cell-based techniques. They hold great potential in the identification of genetic determinants of disease [4], in discovering drugs modifying/modulating cellular functions [5], and in dissecting the complex and dynamic behavior of cells in relation to local environment, especially in the field of stem cells and regenerative medicine [5,6].

In multicellular organisms, cells are complex and dynamically changing systems, with the ability to differentiate and specialize giving rise to different tissues while maintaining a constant genome. These processes are guided and determined by their interactions with the entire microenviroment, composed of other cell types, three-dimensional (3D) extracellular matrix (ECM), and cascades of molecular and physical signals (hormones, growth factors, cytokines and secreted proteins from neighboring cells and tissues). At the same time cells are able to alter the composition of the ECM producing signals affecting other cells or promoting their movement [7]. Since most of this complexity is lacking in standard *in vitro* models, the micro- and nano-technology community is investing major efforts in the artificial replication of cell microenvironments. Bioengineered environments that combine tissue-specific transport and signaling are critical to study development, regeneration and disease in settings predictive of human conditions. With traditional cell culture all these aspects can be analyzed one at a time with a limited chance of parallelization using continuous perfusion bioreactors [8]. As already mentioned, standard cell culture techniques fail to properly mimic the *in vivo* conditions: the relatively large operating volumes and the need of periodic exchange of media do not allow for the generation of precise spatial and temporal patterns of stimulation. As a consequence, soluble growth factors are typically present in poorly controllable concentrations, oxygen concentration is too high, cell-cell interactions are rarely organized and 3D disposition of cells is largely absent. In the area of cell-based biochips, engineers, physicists, chemists, and biologists joined forces to explore the feasibility of cell culture scale reduction, multiplexing, data integration, and adoption of microfluidic technology. In addition, integration with analytics and detector microsystems resulted in new multifunctional tools useful for basic research on cell and tissue biology, as well as for biochemical, biomedical and pharmaceutical research [9]. Some of these tools simply represent miniaturized version of conventional laboratory techniques while others overcome drawbacks of normal 2D cell culture, especially where multiple cell types are grown together mimicking the 3D organization of an organ and the movement of fluids [10]. Further advances leading to increased availability of integrated cell biosensors will allow more efficient monitoring of drug effects together with a reduction of costs and time for actual analyses. When the sensing element is the living human cell, it will also be feasible to abandon the reliance on animal testing. Recently, Neuzi and colleagues reviewed lab-on-a-chip technology for drug discovery [11] highlighting the economic, psychological, legal and technological challenges related to their introduction as substitutes for the well-established traditional methods. From the economic point of view, previous investments in classic instrumentations will be lost in their replacement with on-chip technologies. Moreover, technicians are conservative to minimize the risk of failure and thus rely on well-established techniques and instruments. Nuezi *et al.* predicted that it will take another generation of biologically trained scientists to overcome these challenges and progress further in this field.

**Figure 1.**
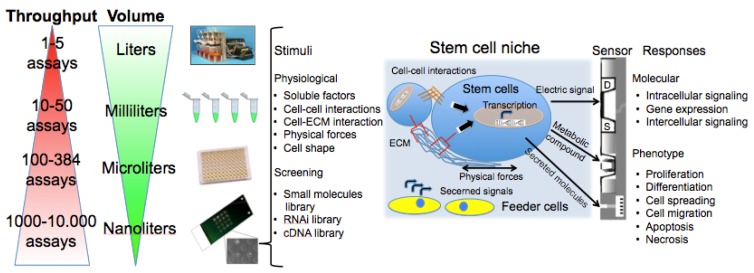
Attributes of a cell-based assay. Left: throughput improvements in laboratory techniques with the dramatic miniaturization of cell assays. With cell microchips, the simultaneous screening of thousands of compounds and different cell responses can be achieved using very small volumes of expensive reagents and small numbers of rare cells. Right: a representation of the stem cell niche (stem cells microenvironment). A list of stimuli and effects (assay variables) involved in the maintenance of stem cell characteristics or in their differentiation are evidenced. Multisensors (e.g., FET with source (S) and drain (D) indicated, and MEA allowing the detection of metabolic and secreted compounds) allow the dynamic analysis of stimuli response in living cells.

Cell-based chips are composed of a bio-receptor or sensing element (receptors on the cell surface or transmembrane channels), a transducer (the cell itself that metabolizes the drug or activates a response to stimuli producing metabolites, current or enzymes) and the true sensor that processes the signal making it readable (Figure 1). This review will describe these components in details, starting from the central element: the cell. We will introduce the cell microarray as a simple, versatile, reproducible, and reliable tool. We will then address some applications for probing cellular differentiation, with particular focus on stem cells since they represent a promise for the treatment of disorders for which there is no effective therapy [12,13]. We will discuss stem cell microelectronic chips differentiating them on the basis of the secondary transducer (the microelectrodes array: MEA, field-effect transistor: FET, light addressable potentiometric sensor: LAPS, electric cell-substrate impedance sensor: ECIS, patch clamp chip, quartz crystal microbalance: QCM, surface plasmon resonance: SPR) and present our results in this field.

## 2. Sensing and Transducer Element: The Cell; Variables and Constants

Cell stimuli are elaborated in an inner area, the cytoplasm, which is separated from the environment by a membrane and a wall in bacteria and plant cells. Cell membranes present pores and receptors, which interact with other cells and the extracellular environment. On the macroscale, cells appear to reside in a stable and homogeneous environment with a spatially uniform ECM, but this is far from the real situation. Cells interact dynamically and communicate with each other through the release of hormones, cytokines and enzymes with highly variable concentrations (in both space and time) in their microenvironment. The dynamicity of the microenvironment allows the adaptation of the cell to the external stimuli [14]. The implementation of this additional layer of complexity requires new tools capable of delivering and recording signals in sub-millimeter and sub-second length and time scales. Next, we will discuss the scale and environment constrains also applicable in stem cell biology.

Figure 1 summarizes assay variables that can be changed among the different elements of cell chips to induce specific cell responses and understand cell adaptation. The most commonly controlled variables are soluble inputs, which can be added or removed from the culture medium. They consist of standardized medium, metabolic substrates, vitamins, antibiotics, unspecified additives derived from animal serum, and of stimuli such as cytokines, growth factors, hormones, and putative therapeutic molecules added at various doses, combinations, and for different periods. Western Blot (WB) or enzyme-linked immunosorbant assay (ELISA) are usually performed to evaluate cell treatment response. Both assays require cell lysis, thus hindering precise and real-time space and time analysis. The major limitation of these techniques is thus the fact that they do not allow time-course studies on the same biological sample. Multiple parallel cultures need to be set up and destructively analyzed at the desired time points, thus increasing sample-to-sample variability and potentially masking other relevant effects. The advent of the Green Fluorescent Protein (GFP) allowed the development of tools for live imaging of cells [15]. It was used to estimate rates of gene expression [16], detect specific cells *in vivo* [17] or as biomarker or biosensor [18]. Even if the discovery of GFP can be considered as a breakthrough for the development of live-cell imaging, quantifications of time-lapse images still need corrections for the auto-fluorescence of culture medium, and methods to track objects and cell movement to identify individual cells without using endpoint nuclear staining or similar.

As shown in Figure 1, cells represent the central element of the sensor. Cells are extremely complex biological entities and incorporate a large number of variables. Thus, particular attention must be paid to the identification of the best cellular response to measure and to the corresponding optimal sensor type capable of detecting it. Examples of successful approaches are the transistor/semiconductor sensors that have been applied to measure ion channel currents [19,20], the analysis of mechanisms underlying both embryonic and adult stem cell functions through printed cell microarray or microtiter plates [21,22], and the analysis of cell-cell interactions with MEA [23] (for a complete review, see [24]).

Particular attention needs to be paid to the intrinsic heterogeneity of a cell population, determined by phenomena such as asynchronous cell division leading to the presence of cells at different cell cycle stages within the same population. Although several methods allow the synchronization cells by morphological features, cellular metabolism or chemical compounds [25], chemical synchronization can potentially disrupt normal cell cycle regulatory processes [26] and the difficulty in elucidating the exact cellular target remains [27]. An implicit assumption of conventional assays is that the measured average response is representative of a typical cell within the population. It is evident how one of the biggest drawbacks of conventional cell assays (WB; ELISA) is the fact that they measure average responses of large cell populations, with the aforementioned heterogeneity potentially obscuring cell-specific responses. For these reasons a single-cell analysis approach is preferable to dissect cell-type specific behaviors avoiding misleading oversimplifications of averaged responses.

Single cell analysis has already been used in gene expression [28–33] or genome analysis (reviewed in [34]). Our group evidenced that in a complex tissue such as skeletal muscle, the analysis of purified single fiber significantly increased the resolution power of the assays [35]. Being a syncytium, the skeletal muscle fiber cannot be properly defined a single cell, nevertheless it represents the functional unit of the tissue. By the use of isolated fibers, we demonstrated that most of information from blood, connective tissue, endothelial and neuronal cells associated to myofibers in the muscle is depleted. This approach can be useful for studies of pathology-altered muscle tissue where cellular heterogeneity is emphasized [36].

Stem cells are unspecialized cells with the ability to self-regenerate and differentiate in many different cell types and this capacity, defined as pluripotency, is the main motivation supporting their intensive study. They play a central role in an organism allowing development, repair of damaged tissue, and cancer that results from stem cell division going awry. Among other research lines, the increased number of donations of cord blood along with the improvements in their storage and maintenance has enabled the possibility to explore new medical therapies based on stem cells. For example, the lengthening of lifetime, life style, and, probably, natural DNA modifications have augmented the probability of undergoing/requiring hematopoietic stem cell transplantation during one's lifetime [37]. Society could benefit even more from this with increases in donor availability and in hematopoietic stem cell transplantation applicability, thus raising the necessity for further knowledge about their biology and use. Stem cells can be divided in embryonic stem cells (ESCs), induced pluripotent stem cells (iPSCs), and adult stem cells (ASCs), and also based on their differentiation potential (Table 1). ESCs are pluripotent cells derived from the inner cell mass of the blastocyst. They grow relatively easily in culture but due to both technical and ethical clues, treatments based on ESCs are limited. An important issue favoring the use of ESCs in regenerative medicine is that they provide a more successful therapeutics than cells taken from older or less healthy donors. This could be associated to longer telomeres [38]. iPSCs are differentiated cells reverted to a pluripotent status through the transfection of specific genes (Sox2, Oct4, c-Myc and Klf4) [39,40]. The genetic manipulation required to obtain iPSC cells is the major drawback for their use in humans' treatment. ASCs are a small population of cells present in adult tissues that are able to differentiate in some particular cell types depending on their tissue of origin. Given their scarce number in adult tissues, they are extremely difficult to isolate. Many researchers are looking at mesenchymal stem cells (MSC) for the treatment of cardiovascular diseases [41] or at adipose derived stem cells [42] for their abundance in adipose tissue. Hematopoeitic stem cells (HSCs) are the best characterized between all the adult stem cells identified. HSCs are located in bone marrow and give rise to all types of blood cells from the myeloid (monocytes and macrophages, neutrophils, basophils, eosinophils, erythrocytes, megakaryocytes/platelets, dendritic cells), and lymphoid lineages (T-cells, B-cells, NK-cells). Stem cell plasticity allows bone marrow mesenchymal stem cells not only the function of forming the hematopoietic microenvironment but the ability of becoming neurons [43], or pancreatic islet cells that are capable of producing insulin [44]. Bone marrow stromal cells (MSCs) can be induced to differentiate in different cells because constituted of an heterogeneous population containing multiple stem/progenitor cell types including mesenchymal stem cells and neural crest stem cells, among others [43]. Based on recent experimental data, a number of clinical trials have been designed for the intravenous (IV) and/or intrathecal (ITH) administration of bone marrow mesenchymal stem cells in multiple sclerosis patients [45]. In addition to general stem cell properties (continuous cell cycle progression for self-renewal and the potential to differentiate into highly specialized cell types) the International Society for Cellular Therapy (ISCT) [46] proposed a more specific panel of markers for the characterization of MSCs. Due to the failure to identify a unique MSC cell-surface molecule, a set of minimal criteria for MSC was recommended, which includes the capability of adherence to plastic surfaces and the expression of the cell surface markers CD44, CD73, CD90, and CD105 with a concomitant absence of CD14, CD19, CD34, CD45, and HLA-DR expression [47].

New tools are becoming available to perform controlled studies on stem cells under conditions that mimic some aspects of the developmental milieu. Here we will discuss some aspects of cell chips applied to stem cells. The components of the stem cell microenvironment that regulate their differentiation include cell–cell and cell–ECM interactions, soluble stimuli and gradients of soluble factors, and the three-dimensional architecture of the niche itself, which shapes and restricts the delivery of these cues. Cell chips help to miniaturize cell culture platforms for parallel analysis to screen, with a systematic and parallel approach, each of the components of the stem cell microenvironment in a compatible scale-dimension (<1 mm).

## 3. Approaches Used for Multi-Testing Cell Responses

### 3.1. High Density Spotting Technique: Cell Microarrays

**Figure 2.**
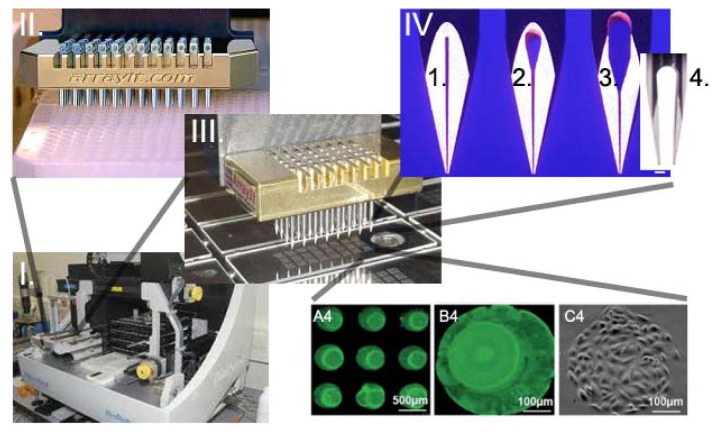
Scheme of spotted-cell microarray spotting. I. Microarray spotter. II. Cell suspension is collected from a 384 well plate by a microarray head supplied with 48 pins. III. Printing on glass slide. IV. Types of printing pins: (1.) pin with a regular uptake channel; (2.) pin with a “bubble” uptake channel; (3.) pin with an “extended bubble” channel and (4.) pin with an enlarged channel suited for cell delivery. Liquid load are 0.25 and 0.60 μL for pin 1 and 2, whereas 1.25 μL are loaded on pin type 3 and 4. Scale bar equals 200 μm. Bottom: enlargement of a printed area in the microarray slide. In A4 are showed nine spots of solution optimized for printing cells with a type 4 pin. B4 shows an enlargement of a spot from A4. C4 shows a spot of printed cells (C2C12 cell line).

Genechip^®^ was developed at Affymax, then Affymetrix, by a team formed by Zaffaroni at the end of the 20th century to be applied in drug discovery processes [48]. A brief overview about microarray uses, companies involved in their market and microarray biosensors is available in [49], while the history of the microarray technology is available in [50]. Based on the key concepts of microarray production (delivery of small volumes of solution, high miniaturization and high throughput assay), the cell microarray was developed. Cell microarrays can be divided in two types: those based on the delivery of cells [51] and those based on the delivery of components (micropatterning [52]) allowing cell attachment or their transformation depending on the cells position on the chip. Cell microarrays inducing cells transformation are typically used to discover gene function [53]. Spotted cells on chips enable the determination of cellular states following exposure to chemical or genetic perturbations. Differently from living cell microarray, in spotted cell chips cells are first treated using standard cell culture conditions and then printed onto glass slides before being fixed. Technical challenges in the production of cell printed microarray are represented by the pins used to print and the capacity to maintain cells in suspension. This last problem was solved by optimizing the viscosity of the suspension medium, while pins were adapted to have slots compatible with cell dimensions (Figure 2) and to avoid clotting and cell shear stress [51]. Microarrays for cell delivery systems allow the analysis of multiple cell types and multiple growth and treatment conditions on a single slide; however, when compared to transfected cell array [53,54], they do not allow the high throughput screening of libraries (e.g., siRNA libraries [53]) or to directly work with live cells on chips [55,56].

#### Genome wide screening

High throughput screening of gene function is of primary importance, especially after the publication of the human genome [57,58]. This event pushed the development of new sequencing technologies (next-generation sequencing is reviewed in [59]) making the whole genome analysis a more affordable task and extending gene identification also in non-model organisms.

The ability to produce libraries of interfering RNA (RNAi) through chemical synthesis [60] or by enzymatic digestion of long double stranded molecules (esiRNAs) [61] allows the selective silencing of practically every gene of an organism. This ability, associated with fluorescence microscopy, provides a uniquely detailed phenotypic readout of cultured cells to discover gene function. In fact, RNAi is a post-transcriptional method of gene silencing in which double stranded RNA mediates the sequence specific degradation of mRNAs. RNAi using cell microarray (reverse transfection), as opposed to RNAi on plate assay, has the advantage of miniaturization and therefore an enhanced throughput. Moreover, cell microarrays allow the delivery of complex stimuli such as concentration gradients, which are hard to generate in plate-based screenings. Furthermore, miniaturization allows the sparing use of RNAi reagents and rare cell lines such as adult stem cells. Gene function can be understood also by the transfection of libraries based on recombinant plasmids and viral vectors. In both cases gain-of-function can be tested as well. Cell microarray associated with cDNA printing was used for the identification of drug targets or to discover the gene function suggested by altered cellular physiology [54]; even if the overexpression of a specific gene may cause an altered phenotype confusing its real function in normal cell. Transfection of cDNA through cell microarray was also used in the characterization of the regulative elements of cAMP-dependent protein kinase [62] demonstrating the feasibility of high throughput approaches in transcriptional regulation. Loss-of-function was first used to select functional siRNA against MyoD gene [63], since not all RNAi sequences are equally efficient in the down regulation of the target gene. This technique was applied in many other studies to characterize different pathways (p53 pathway [64], human proteasome [65] and NF-kB pathway [66]) or in the *D. melanogaster* cell analysis [67,68]. Not only RNAi and recombinant plasmids were used in cell microarrays but also the spotting of small molecules was applied in order to monitor their effects. Spotting of small molecules to test with cell microarrays was obtained by embedding them into biodegradable materials to avoid diffusion [69]. In the next paragraphs we will discuss the advances of the cell microarray technology focusing on stem cells.

*Stem cell microarray* Reverse transfection was used not only with somatic cells, but also with stem cells [70]. The increasing importance of these cells in regenerative medicine make essential the comprehension of molecular mechanisms involved in the maintenance of pluripotency and in the activation of differentiation. In this context, RNAi represent a powerful strategy for the discovery of gene function. Yoshikawa *et al.* demonstrated the feasibility of loss-of-function approach in human mesenchymal stem cell [70]. Interestingly, transfection on solid surface is affected by the deposition of an ECM protein in conjunction with DNA to be transfected [70]. ECM proteins regulate cell signaling interacting with cell receptors and integrins [71], but they can also act as microenvironment determinant establishing availability and gradients of growth factors [72]. Moreover, domains in ECM fibrils (e.g., EGF-like domains) may act as ligand for EGF receptor to trigger specific signals. The ECM degradation itself, obtained through lytic enzymes or metalloproteinases, results in the release of either EGF-like domains or of the ECM-linked growth factors making them available in a cell specific microenvironment. We previously proved that the cell microenvironment and the substrate elasticity are fundamental determinants for the behavior of adhering C2C12 cells (an embryonic muscle cell line). Cell behavior is dependent on the spotted ECM protein [73] and also on physical/mechanical stimuli conveyed by ECM stiffness [74,75]. Among the pioneering works in this field we should cite two publications from the Langer [76] and Bhatia groups [22], reporting important data for the comprehension of the ECM-cell interactions. They characterized the stem cell behavior in relation to different contact surfaces. In particular, Anderson *et al.* [76] studied 1,700 human ESC-material interactions using a cell microarray, obtaining results that were impossible to achieve with classical screening methods that require high quantities of cells and materials. Moreover, they demonstrated that certain monomers inhibited ESC attachment or spreading, thus excluding their use in the production of ESCs-populated scaffolds. Flaim *et al.* [22] analyzed a different aspect of stem cell physiology, but still related to their niche. 32 different combinations of five ECM molecules were analyzed for their ability to allow survival and differentiation of ESCs. Collagen IV best allowed the maintenance of primary rat hepatocyte phenotype, while a mixture of laminin, collagen I and fibronectin allowed a better differentiation of mouse ESCs toward a hepatic fate.

Recently, it was evidenced that 80% of the genome has some biochemical function (ENCODE project [77]) and that 2,000–5,000 human genes out of the 30,000 in the genome are predicted to enter in the secretory pathway [78]. This result dramatically increased the number of possible combinations of the ECM components that was recently used by Brafman *et al.* [6] to identify conditions that promote human pluripotent stem cells (hPSC) attachment and growth. hPSCs constitutively expressing GFP were cultured on arrays using a variety of conditions demonstrating the possibility to apply this technology also to living cells.

Differentiation of ESCs is typically obtained with the employment of embryoid bodies (EBs) [79], addition of cytokines to culture media [80] or co-culturing with feeder cells [81,82] (for a review, see [83]). The co-culture method seems to be the most efficient [84], but it is dependent on feeder cells, among others. Cell microarrays were used for screening feeder cells for ESC differentiation [85]. Fibronectin was used to allow feeder cells pattering and only spots carrying PA6 cells (and not HUVEC and COS-1 cells) induced neural differentiation of ESCs. These applications demonstrate the flexibility of cell microarray technology and the possibility to integrate different information recovering fundamental data in ESCs survival, duplication and differentiation.

### 3.2. Micropattering: Microengineering Meets Cell Biology

**Figure 3.**
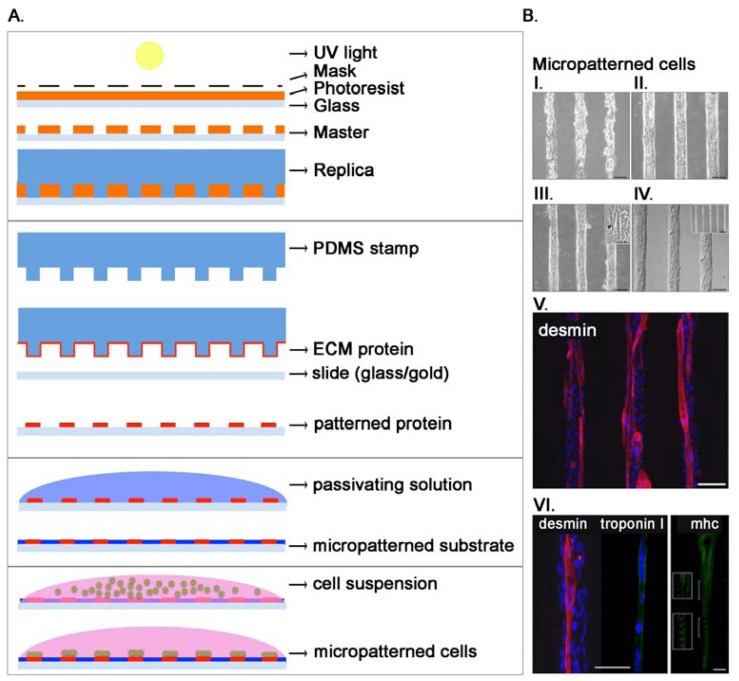
(**A**). The soft lithography method. PDMS (polydimethylsiloxane) stamps are formed by replica molding onto a negative photoresist mold, generated via UV-mediated (ultraviolet) selective crosslinking through a photomask containing the desired features of photosensitive resins. A PDMS stamp is used to transfer ECM onto the supporting materials, creating a specific pattern for cell adhesion. (**B**). Satellite cells cultured on patterned hydrogel. 5 h after seeding, mouse satellite cells are attached only in correspondence of the laminin lanes (I.) producing aligned pattern after 3 (II.) and 7 (III.) days in culture (scale bar = 100 μm). The inset in (III.) shows the occurred fusion into myotubes (arrow; scale bar=37.5 μm). Interference microscope image shows aligned satellite cells after 7 days in culture (IV.) (scale bar = 100 μm). Newly formed myotubes express desmin (V.) (scale bar = 100 μm), troponin I, and mhc (VI.) (scale bar = 75 μm and 25 μm respectively for troponin and mhc). Organization in regular and uniform striations of mhc is highlighted on the two insets (VI.). Cell nuclei were counterstained with Hoechst (blue). Modified from [96].

As stated before, cells live within a complex microenvironment that plays a crucial role in normal and pathologic conditions. In order to use cultured cells as models for tissue processes, researchers have to design complex patterns and structures able to mimic the *in vivo* microenvironment in an *in vitro* setting. Microfabrication technology helps in the production of these tools allowing the culture of cells on well-defined surfaces, patterning of cells into defined geometries, and measurements of force associated with cell-ECM interaction. Self-Assembled Monolayers (SAM) are used with surfaces such as gold [86] while metal evaporation with specialized masks, initially used in mid-1990s [87,88], allows the production of specific adhesive areas for living cells on non-fouling backgrounds. This method is however not easily adaptable for general uses in biological research. Some years later, the advent of microcontact printing and soft lithography allowed the production of chips competent for cell adhesion only in definite areas [89,90]. Initially, through photolithography, a mold with an array of micrometer-sized features was designed and used to produce a complementary elastomeric stamp (usually polydimethylsiloxane, PDMS). Stamps can be inked with silanes, alkanethiols (covalently linked to gold) or with ECM proteins [91–93] and used to transfer the inked material to the receiving surface (Figure 3(A)). Micropattering can define cell adhesive regions with a 50 nm resolution that is limited by the method used to generate the mold. These techniques have been largely used, and in our groups mainly to study skeletal muscle cells [75,94–96]. The production of functional cardiac and skeletal muscle tissues is certainly a challenging task, since they are composed *in vivo* by a complex aggregate of cells strictly associated and communicating through gap junctions in the heart [97] or forming a syncytium in skeletal muscle [98]. We evidenced how microcontact printing of ECM proteins allows the orientation of single muscle cells and the formation of mature and functional myofibers [96] (Figure 3(B)). The tool can be used for pharmacological or biological studies at the single fiber level. Moreover, we demonstrated that electrical stimulation in association with cell orientation is able to improve differentiation of muscle cells [95]. It is now widely assessed that the mechanical properties of the substrate where cells adhere greatly influence and guide cell proliferation and differentiation [99,100]. To print adhesion proteins for myoblast cells adhesion we used a thin film of photo cross-linkable elastic poly-acrylamide hydrogel because of its physiological-like and tunable mechanical properties (elastic moduli, E: 12, 15, 18 and 21 kPa). We demonstrated that substrate stiffness regulated the extent of myotubes formation and maturation, with higher percentages measured on substrates with *in vivo*-like stiffness [75]. We also modulated the spatial organization of cells demonstrating that wider adhesion lanes showed a decrease in murine myoblast proliferation while fusion index increased in narrower lanes. Our results underline the role of micropatterning in shaping the cellular niche through the accumulation of secreted factors [94]. Patterned surfaces have also been used to investigate the effects of cell–cell contact in a well-controlled fashion. Traditional methods are based on seeding cells at different density, but this method produce cells with different sizes and shapes in function of the density. Using a bowtie-like pattern Nelson positioned two cells near each other to demonstrate that cell-cell contact lead to a decrease in cell spreading and proliferation, but not if cell spreading is kept constant [101]. Not only homotypic cell interactions were inspected using micropatterning but also heterotypic, being these latter critical for liver and breast functions. For example, Collagen I was deposited in a controlled pattern using photolithography demonstrating that primary hepatocytes cultured in adjacent lines with fibroblasts increase their capacity for urea and albumin secretion [102].

*Stem cell pattering* Micropatterning allows precise control of the shape of cell-adhesive islands on a substrate, a known important determinant of stem cell fate. An example was provided by Wan's work [93]. Human adipose stem cells were cultured in differently shaped micropatterned adhesive surfaces, demonstrating a correlation between proliferative condition for more spread cells and differentiative for smaller and elongated cells. We confirmed a similar behavior for human murine satellite cells (mSC). Seeding multipotent muscle cells onto organized rectangular micropatterned polyglycolic acid scaffold allowed a better myoblasts differentiation [103], holding great importance for cell therapy for skeletal muscle disorders. Like cell microarrays, stamp-based micropattering has also proven to be an important technique to examine how cell-substrate interactions influence stem cell proliferation and differentiation, among other phenomena. Cell shape and topographical features dictate cell behavior allowing stem cell lineage commitment (Table 2).

In summary, to test how topological features influence cell behavior soft lithography is particularly advantageous thanks to its flexibility in creating patterns with different geometries. A drawback of this technique is that it has to be used with adherent cells. Moreover, 2D micropatterns tends to deteriorate over time [104]. Understanding signals that define stem cell niche can be improved by 3D culture approaches. Exploiting bioengineered scaffolds and nanoscale devices mimicking the mechanical properties of natural tissue would offer new tools for approaching cells spatial organization, differentiation and tissue synthesis.

### 3.3. 3D Cell Culture and Tissue Organization

3D cell culture models have recently gathered great attention because they can promote cell differentiation, organization and tissue-like distribution that cannot be attained with conventional 2D systems. Recent reviews addressed new advances in 3D culture that leverage microfabrication technologies from the microchip industry and microfluidic approaches to create cell culture microenvironments that both support tissue differentiation and recapitulate the tissue–tissue interfaces, spatial-temporal chemical gradients, and mechanical microenvironments of living organs [10]. Here we will focus our attention on the 3D stem cell culture not discussed in [10].

Among other materials, hydrogels can be used for the formation of 3D structures, even though the bigger the scale, the more difficult it gets to control 3D architecture and cell-cell interactions. Moreover, it is hard to replicate the actual complexity of *in vivo* tissues. Microscaled hydrogels have none of these limitations and in contrast allow minimizing diffusion limitations while maintaining tissue-like microarchitectures [112]. Microgels can be manufactured by micromolding, emulsification, photolithograpy and microfluidic techniques. Advantages and disadvantages of each method are summarized in Table 3. The association of monomers composing the gel and crosslink agent determines the mechanical, physical and biochemical characteristics that in turn influence stem cells behavior [113,114]. Yeh *et al.* demonstrated that methacrylated hyaluronic acid or poly(ethylene glycol) diacrylate could be used to produce hydrogels embedding mouse ESCs [115]. Cells spatial distribution was controlled via the micromolded stamps shape, and the technique was used for fabricating 3D microcultures. Their constructs are compatible with most immunofluorescence methodologies and most microscopy detection techniques.

Significant improvements in the field of stem cell culture and tissue regeneration should include innovative culture systems that integrate sophisticated monitoring platforms to ensure continuous culture evaluations at a cellular level. For this purpose micro- and nano-biosensors constitute promising solutions. Once integrated in the bioreactors they would be able to regulate cell culture parameters closing the feedback loop between measured values and corresponding variations in culture conditions.

**Figure 4.**
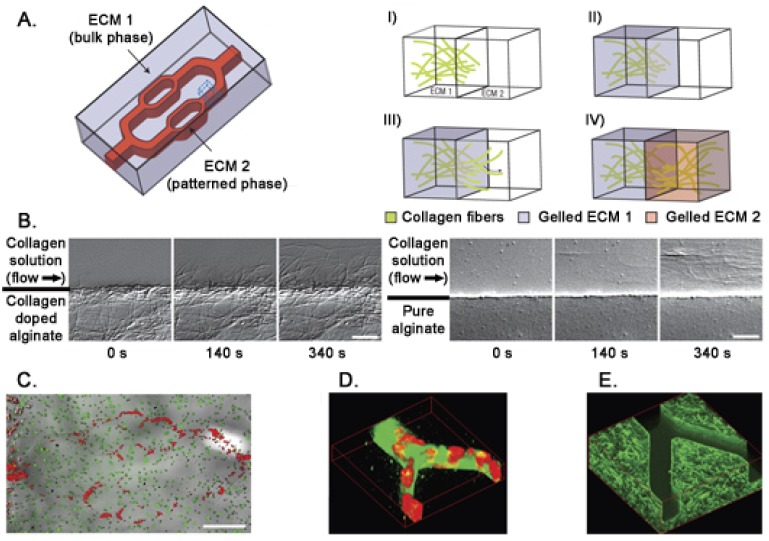
**A.** Schematic diagram of a construct consisting of multiple 3D matrices: a microfluidically patterned phase and a bulk microfluidic hydrogel phase. Magnified view of the interface (boxed region in (**A**)) showing the formation of each phase. (I) The bulk phase is formed by doping collagen into an alginate solution and allowing a collagen fibers network to form (by increasing temperature). (II) The alginate is gelled (by ionic crosslinking) around the collagen fiber network to complete formation of the bulk matrix. (III) A second collagen-doped ECM (for example, fibrinogen) solution is then patterned within the bulk phase. As temperature is increased, collagen precursors in the second ECM nucleate and assemble from exposed collagen fibers at the interface to integrate the two matrices. (IV) Formation of the patterned ECM is completed on gelling of fibrin in this example (by diffusion of a thrombin solution into the construct to cleave fibrinogen into fibrin *in situ*). (**B**). Time-lapse differential interference contrast imaging of collagen fibers assembly at the phase interface. Collagen fibers in the patterned ECM assemble from the collagen-doped bulk phase interface into the polymerizing ECM solution (left panel), but do not nucleate from a pure alginate bulk phase interface (right panel). Scale bar is 10 μm. (**C**). HUVECs (red) are localized to the channel pattern, whereas the fibroblasts (green) are distributed uniformly throughout a pure alginate bulk phase. Scale bar is 500 μm. (**D**) and (**E**). Confocal reflectance microscopy. In (D), the 3D reconstruction of microfluidically patterned collagen (green) seeded with HUVECs (red) in a bare alginate bulk phase confirms that HUVEC-seeded collagen completely filled the channels (as opposed to coating the walls) and that the phases were separated by the intended sharp boundaries. In (E), the 3D reconstruction of the confocal *z* series through a collagen–alginate bulk phase before microfluidic patterning of collagen. Modified from [116].

In living tissues the microvascular system modulates the concentration of soluble molecules such as metabolites, gases, therapeutics, and anti-fouling agents. To mimic this functional structure *in vitro*, microfluidic gels can be used. Photolithographic techniques allow the formation of channels on a 10–10^3^ μm scale implementing physiological fluids movement in synthetic biomaterials. Moreover, microfluidic flow of ECM precursors and cell suspensions within the hydrogel bulk phase allows the formation of stable patterns of different 3D extracellular matrices interfaced with cell cultures [116] (Figure 4). An alternative for a better alignment of micropatterned protein structures and cells is dielectrophoresis [117]. Cells are moved in a heterogeneous electrical field across the hydrogel allowing their accurate positioning inside the 3D structure. Drawbacks of dielectophoresis are related to the use of buffers that are potentially toxic and to the presence of relatively strong electrical fields that induce heating of the solution [118]. Here we don't extensively discuss the production of 3D hydrogels (for more details see [119–122]), instead we focus on their applications in stem cells analysis.

3D polymeric scaffolds uniformly populated with stem cells can be implanted in injured tissues to promote healing. We demonstrated that micro-patterned scaffolds seeded with murine satellite cells and implanted in injured mouse skeletal muscle allow a better deliver of satellite cells than direct cell injection [103] and that constant bioreactor-driven perfusion of nutrients improves cell density and distribution throughout the scaffold [123]. These advantages derive from the improved spatial cell organization and dense cellularization within the scaffold combined with the effect of fresh medium perfusion mimicking blood circulation. Other important applications are the ability to differentiate stem cells in pancreatic islets [124], neuronal cells [125], and vascular grafts [126]. Cardiovascular diseases are one of the major problems in the developed society (*i.e.*, atherosclerosis [127] or heart failure [128]), while the ability to recover neuronal functions in degenerative pathologies such as Alzheimer or Parkinson's diseases is still a challenging problem. As previously discussed, one of the characteristics that make stem cells research of paramount importance is their ability to recapitulate a diseased or injured condition. In this sight, it is fundamental to be able to expand them while preserving their ability to differentiate. The obtainment of high cell numbers is especially difficult for adult stem cells, so their efficient expansion become a crucial step for therapy.

## 4. Integration of Microelectronics and Cells

Microelectronic cell-based biosensors have the potential of providing rapid, sensitive, low-cost measurement technology. Cells are naturally equipped with a host of receptors that can transduce chemical and biological signals into electrical ones. The on-off behavior of cellular receptors and ion channels induces the migration of charged proteins and ions on both sides of cellular membrane, which could be in turn coupled with microelectronic devices. These sensors can be applied to measure extracellular action potentials, impedance, and transmission paths of ionic channels detecting, for example, the transmission velocity of biological signals along layers of neurons. However, successful culture of cells on microelectronic devices is still a challenging issue. The main problem is that the material itself is not attractive to cells in terms of roughness, hydrophilicity, surface functional groups, and stiffness. Further work is needed to improve the surface characteristics of transducers. According to the transduction method, microelectronic cell-based biosensors can be of different nature: microelectrode arrays (MEA), electric cell-substrate impedance sensor-based (ECIS), field-effect transistors-based (FET), light addressable potentiometric sensors-based (LAPS), patch clamp chips, surface plasmon resonance chips (SPR), and quartz crystal microbalance chips (QCM). Here we will discuss their structure and applications in particular related to stem cells.

### 4.1. Microelectrodes Array: MEA

**Figure 5.**
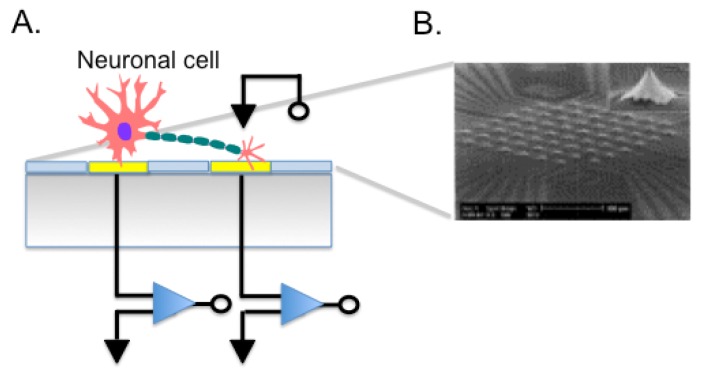
(**A**) Schematic diagram of MEA cell-based biosensor. In yellow the electrode and in blue the insulator. (**B**) SEM picture of 3D MEA recording area. It is composed of 60 tip-shaped protruding platinum electrodes. The height of the glass tips is about 60 μm. Modified from [132].

MEAs are fabricated by depositing Au, Ir, Pt, or other metals on silicon substrates or glass to form electrodes, connecting leads, passivation layers, and forming electrode sites where the cells or tissues contact (Figure 5(A)). Given their relatively simple fabrication and good biocompatibility, they have been used in many applications such as cell pattering [23], drug screening, observing signal transfer of cardiac myocytes [129] or to evaluate ion signals in neuronal cells [130]. For instance, we employed a microelectrode array to perform single cell experiments. We cultured the K1 subclone of Chinese Hamster Ovary cells (CHO-K1) onto the chip surface demonstrating the feasibility of single cell transfection. Using an on-chip-single-cell electroporation protocol, we transformed cells adherent to electrode with specific molecules [131]. We were able to modulate the permeability of the cell membrane, which represents a step towards a high throughput gene analysis on single cells. MEA technology still faces some problems. For example, substrate surface is easily eroded when dipped in the culture solution for a long time and the gap between cells and electrodes is difficult to control during cell seeding also in case of cell movements after adhesion to the MEA. Cell positioning with respect to the electrodes affects measures. Other than planar microelectrodes, also 3D electrodes were used (Figure 5(B)), and electrodes shaped as microtips allowed to record signals deeper in the cell layer [132].

#### Stem cells onto MEA chips

Communication between cells in the nervous system is fundamental for all the complex functions that are provided by this tissue. Pathologies of the neuronal cells are particularly debilitating and understanding the regenerative capacity of neuronal cells is challenging. Using a co-culture approach onto MEA chip, Stephens *et al.* demonstrated the ability of neural progenitor cells to generate super bursts of activity, which is usually only found in a developing mammalian brain [133]. Their chip allowed monitoring a network of cells studying what happens when new stem cells are added and how many cells will be needed to restore brain function. The growth of human neural networks of stem cells on a MEA was studied also by Pizzi *et al.*, demonstrating an organized response after stimuli [134]. The chip can be used both to stimulate cells and to record responses to stimuli, as in drug discovery screenings. For example human embryonic stem cell derived neuronal networks were used in neurotoxicological screening during drugs exposure [135]. Of great relevance is the issue of cardiac and hepatic drug toxicities. To address this problem, Mummery's lab implemented the use of patch clamp analyses and MEAs on human cardiomyocytes derived from hESCs, used as a renewable and scalable cell source more closely resembling functional cardiomyocytes of the human heart [136]. Their system was validated for the capacity of performing reliable cardiac safety pharmacological assays. Field potential duration (FPD) values following exposure to different drugs could be recorded, and drug-induced QT changes in response to selective ion channel blockers were measured, highlighting adverse effects of the tested drugs with greater confidence than standard *in vitro* assays.

Since mesenchymal stem cells (MSC) can differentiate into multiple tissue-specific cells (adipose, bone, tendon, cartilage, muscle, and marrow stroma [137]), their high throughput characterization would be of a great benefit for use in regenerative medicine. Cho *et al.* demonstrated that platinum electrodes-based chip can be used for the characterization of hMSCs growth during long-term cultivation [138].

### 4.2. Electric Cell-Substrate Impedance Sensor: EICS

**Figure 6.**
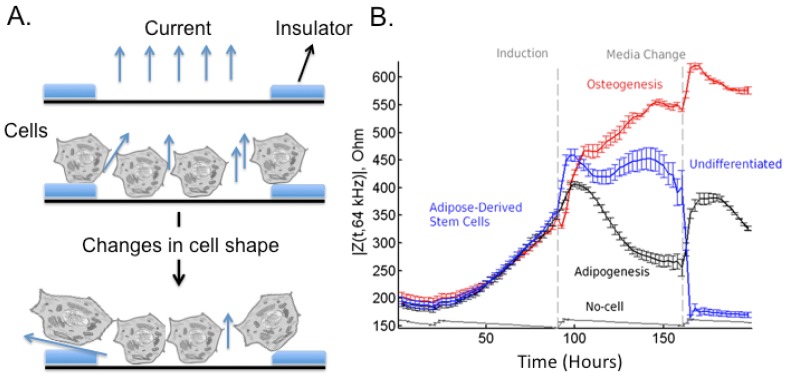
(**A**) Schematic diagram of EICS cell-based biosensor. Measuring the current and voltage across a small empty electrode, the impedance, can be calculated. When cells cover the electrode the measured impedance changes because the cell membranes block the current flow. (**B**) Time-course measurement of mean impedance at 64 kHz. Adipose derived stem cells (ADSCs) were seeded (*t* = 0) on multiwell preprinted electrodes arrays. At *t* = 93 h, ADSCs were induced toward osteoblasts (*n* = 3) and adipocytes (*n* = 3) with osteogenesis and adipogenesis differentiation medium, respectively. Non-induced ADSCs (*n* = 3) were kept growing after confluence until cell detachment occurred. Clear differences in impedance can be observed between all groups. Modified from [140].

Stem cell differentiation was also studied using EICS sensors (Figure 6(A)), which allow investigating bioelectrical properties of cells. The most important components of EICS sensors are the frequency characteristics and sensitivity that can be attained; in this sight, potential problems might be related to the obtainment of sufficient sensitivities though optimized design of the electrodes. For an exhaustive dissertation about these problems see [139]. Briefly, in ECIS a small alternating current (I) is applied across the electrode pattern at the bottom of the ECIS arrays resulting in a potential (V) across the electrodes. When cells are added to the ECIS arrays and attach to the electrodes, they act as insulators increasing the impedance (Ohm's law Z = impedance = V/I). When cells are stimulated to change morphology or proliferate, the capacity to cover the electrode changes with the electrode impedance.

hMSC differentiation was monitored using EICS sensor [141], and the maximal resistance values of the hMSC layer in the ECIS assay correlated with the degree of neural differentiation. Also, adipose-derived stem cells have been intensively studied for their ease of isolation in high concentration from lipoaspirates [142] and for being a realistic source of autologous stem cells. Bagnaninchi *et al.* [140] used the impedance monitoring to follow differentiation of adipose-derived stem cells into osteoblasts (Figure 6(B)), proving that methods for quantitative monitoring of adult stem cell differentiation could contribute to the automation of stem cell culture, the optimization and design of defined media and substrates. Other analyses were based on EICS to verify the adhesiveness of stem cells, for example in response to paracrine stimulation [143]. EICS is a label-free and noninvasive monitoring technique, a characteristic of paramount importance in stem cells characterization since most other tools end up being invasive and precluding their therapeutic potential.

### 4.3. Field-Effect Transistor: FET

**Figure 7.**
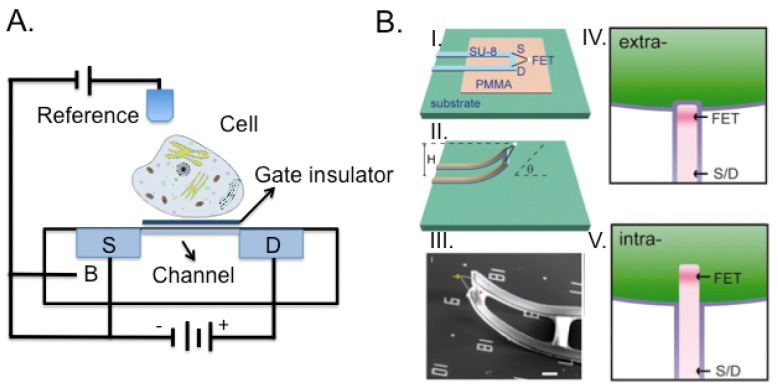
(**A**) Cell/transistor hybrid. The open-gate area of the FET is completely covered by one cell as indicated in the schematics. S and D designate the built-in source and drain connections, while B the bulk. (**B**) Schematics of 3D device fabrication (I. and II.) The dimensions of the lightly doped n-type silicon segment (white dots) are ∼80 by 80 by 200 nm^3^. *H* and θ are the tip height and orientation, respectively. In III the SEM image of an as-made device. Scale bar 5 μm. Highlight of extracellular (IV.) and intracellular (V.) nanowire/cell interfaces. Modified from [144].

Field effect transistors were first patented in 1925 by Julius Edgar Lilienfeld. The device consists of an active channel through which charged carriers flow from the source to the drain (Figure 7(A)). Source and drain terminal conductors are connected to the semiconductor through ohmic contacts that allow the formation of a linear and symmetric current–voltage(I-V) curve. The conductivity of the channel is a function of the potential applied across the gate and source terminals.

In 1970 Bergveld first employed the metal free gate FET for extracellular ion concentration measurements (ISFET) [145]. Since then, several FET modifications were developed such as enzyme modified (ENFET), immune-reaction based (IMFET), and chemically sensitive (CHEMFET). For a review of the development of FET in biological area and specific discussion of cellular signaling see [146] and [147], respectively. CHEMFETs allow measurements of different signal parameters such as extracellular pH, concentration of ions, redox potentials, oxygen consumption and CO_2_ production. FET are also used to quantify the extracellular potential of electrogenic cells (e.g., neuronal and muscle cells) [148,149]. Recently, arrays of ISFET on CMOS electronic chips were introduced to improve parallelism and throughput in next generation sequencing [150]. The advantages deriving from the use of FET to monitor cell behavior are: fast response of the sensor, low cost and non-invasive long-term recording processes. Similarly to MEA, the distance between the detector and the cells has a strong impact on the sensitivity of FET-based detection.

Rather than forcing the cell to adapt to the substrate, Tian *et al.* developed a 3D device [144], composed by a movable nanoFET where the source and drain electrical connections could be moved into contact with the cell and probe within the cell membrane. We evidenced how the implementation of the third dimension to integrated circuits technology improved performance and functionality [151]. 3D integration provides major advantages as compared to standard chips. In fact, being able to distribute on different chips the sensitive low noise analog circuits in low-noise operation from the digital circuits, would lead to improved sensitivity performance and space exploitation. Recently, we addressed the issues related to processing and material solutions to accomplish the robustness requirements towards prolonged contact with electrolyte solution and surface cleaning processes [152,153].

Nano-objects such as nanowires [154] and carbon nanotubes [155,156] have received increasing attention. Nanowires represent a class of inorganic materials that are surface-passivated by thin oxide layer and serve as electrodes or connecting bridges between micro- and nano-electronic devices. Carbon nanotubes exhibit useful properties such as mechanical strength, enormous surface area and large-scale high density. However, the extreme sensitivity of nanowires- and nanotubes-based field-effect sensors is hampered by their sensitivity to impurities and other ionic species in the analyte solution. Nonetheless, 3D structures with nanowires will allow fast drug discovery in a more suitable cell environment (Figure 7(B)). As an example, Tian *et al.* [154] developed this idea using nanowires meshes not only to allow cell to grow in a 3D structure but also to act as a sensor measuring changes in the beating frequency of a heart patch, following exposure to specific drugs.

### 4.4. Light Addressable Potentiometric Sensor: LAPS

**Figure 8.**
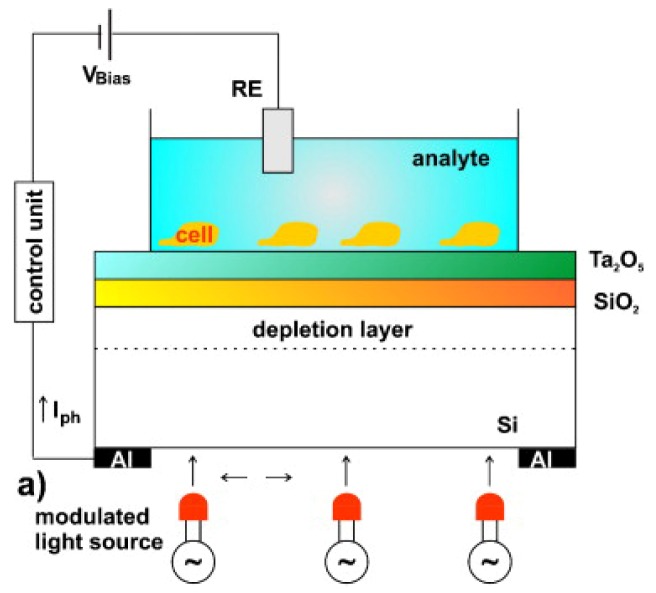
Schematic set-up of a LAPS device with living cells and light sources. Modified from [147].

LAPS were first proposed in 1988 by Hafeman *et al.* [157] (Figure 8). Most researchers using LAPS adopted the commercial microphysiometer produced by Molecular Device Corporation [158] and use them also in the analysis of single cell response [159]. LAPS can be used both to monitor extracellular potentials, such as in FET and MEA chips, and for cell metabolism analysis. The main difference between these techniques is that the measuring sites with LAPS are not predetermined, while the opposite is true for FET and MEA arrays. Every event that induces variations in the surface potential can be detected (like in the ISFET). Given that in LAPS chips a light exciting the structure silicon/silicon oxide/silicon nitride creates the measurable surface potential currents, the resolution is correlated to the capacity to illuminate a particular region of the sensor. LAPS was used in different studies to analyze cells' electrophysiological properties [160], signaling mechanisms [161], ligand-receptor binding [162], and drug analysis [163]. Here we will address some applications with stem cells. Liu *et al.* used LAPS in the study of cardiogenic cells [164], monitoring embryonic stem cells differentiation into cardiomyocytes, although without thoroughly investigating stem cell behavior. This study is important because is setting the ground for the development of a platform to evaluate cardiotoxicity of new drugs [165]. Mouse embryonic stem cells cultured on the surface of LAPS were induced to differentiate into synchronized and spontaneously beating cardiomyocytes. Since changes of extracellular potentials and cell shape during contractions induce modulation of photocurrents in the LAPS system, it was possible to record the prolongation of ventricular action potentials induced by drugs and correlated it with cardiotoxicity [165]. Moreover, the sensing of intracellular biomolecules, enzyme activity and pH in real time (easily allowed by LAPS) can contribute for a better understanding of biological processes in stem cells leading to the development of strategies to control and use them therapeutically.

### 4.5. Surface Plasmon Resonance Chip (SPR) and Quartz Crystal Microbalance Chip (QCM)

Mechanisms involved in cell attachment can be analyzed through the use of SPR technique, an optical-electrical phenomenon arising from the interaction of light with a metal surface, enabling the detection of the presence of a biopolymer on chemically modified gold surfaces. The working principle is the change in the local refraction index upon adsorption of light. SPR could be used in association with electrochemistry (EC-SPR) where the thin metal film on the substrate is used not only to excite surface plasmons, but also acts as a working electrode for electrochemical detection or control. SPR, in association with different surface functionalization, may be used to obtain distinct spectra for specific cell types. This technique was used to analyze mesenchymal stem cells [166,167]. Kuo *et al.* [167] used the adhesiveness of stem cells to the sensor surface through OB-cadherin, which is expressed during osteogenic differentiation and thus is a good target for the sensor in evaluating osteogenic differentiation of MSCs.

The quartz crystal microbalance (QCM) is a very sensitive sensor capable of detecting small mass changes based on the piezoelectric effect. Some properties of cultured cells had been successfully monitored with QCM, such as cell attachment, proliferation, and cell-substrate interaction. Although this technique is not widely applied for stem cells studies [168,169], Pirouz *et al.* [169] used it to evaluate the ability of mesenchymal stem cells to adhere to surfaces. This paper is important for its role in assessing the issue of stem cell-substrate interaction, which (as discussed above) is fundamental for the understanding of cell differentiation processes. Pirouz *et al.* [169] used a modified QCM: the QCM-D. This technique can simultaneously measure the oscillation frequency of the quartz and the dissipation energy of the oscillating system providing more useful information about cell-surface interactions for biomaterials. The QCM-D sensor is commercially distributed by Q-Sense AB (Sweden).

### 4.6. Integration of Different Sensors and Microfluidic Approaches: Future Perspectives on Single Cell Analysis

Each different microchip is able to detect specific responses (Table 4) with different principles and sensitivity. Nowadays the integration appears a challenge for the comprehension of cell processes and to dissect processes guiding stem cell proliferation and differentiation. Moreover, in the field of drug discovery, before using a drug in time consuming and expensive experiments (e.g., using animal models) a precise screening of different drugs and their effects on different cells is needed. Integration of multiple sensors with microfluidic platforms appears a promising way in the development of new biochips. A recent review of Neuzi *et al.* [11] discussed the application of the lab-on-a-chip concept in drug discovery. Here we will discuss its integration for stem cells analysis.

There are three types of integration: (a) integration of the same or similar sensors with the same or similar functions; (b) integration of different sensor elements with different functions; (c) integration of different chips that can monitor the different parameters in different detecting environment [170–172]. Microfluidic platforms are relatively easy to integrate in all types of microchips because of their ease of fabrication and low cost of raw materials, with the consequent reduction of experimental costs and the capability to produce controlled microenvironments and stable concentration gradients [173,174]. The differentiation of hMSCs in adipocytes was followed in a microfluidic chip demonstrating that it depends on the initial cell density (stem cell niche) with a relation between cell density and differentiation rate [175]. Drawbacks of the microfluidic approach are that PDMS, the material usually used to fabricate channels, has an affinity for small hydrophobic molecules and thus could lead to biomolecule absorption/adsorption from the medium. Moreover, the permeability of PDMS to water vapor can also lead to media drying and thus change its osmolarity.

**Figure 9.**
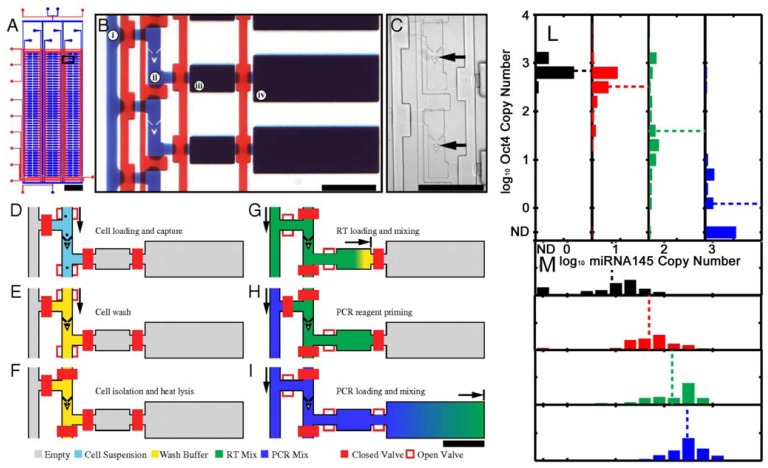
(**A**) Schematic of microfluidic device. Scale bar: 4 mm. The device features 6 sample input channels, each divided into 50 compound reaction chambers for a total of 300 RT-qPCR reactions using approximately 20 μL of reagents. The rectangular box indicates the region depicted in B. (**B**) Optical micrograph of array unit. For visualization, the fluid paths and control channels have been loaded with blue and red dyes, respectively. Each unit consists of (i) a reagent injection line, (ii) a 0.6 nL cell capture chamber with integrated cell traps, (iii) a 10 nL reverse transcription (RT) chamber, and (iv) a 50 nL PCR chamber. Scale bar: 400 μm. (**C**) Optical micrograph of two cell capture chambers with trapped single cells indicated by black arrows. Each trap includes upstream deflectors to direct cells into the capture region. Scale bar: 400 μm. (**D–I**) Device operation. (**D**) A single-cell suspension is injected into the device. (**E**) Cell traps isolate single cells from the fluid stream and permit washing of cells to remove extracellular RNA. (**F**) Actuation of pneumatic valves results in single-cell isolation prior to heat lysis. (**G**) Injection of reagent (green) for RT reaction (10 nL). (**H**) Reagent injection line is flushed with subsequent reagent (blue) for PCR. (**I**) Reagent for qPCR (blue) is combined with RT product in 50 nL qPCR chamber. Scale bar for *D*–*I*: 400 μm. (**L** and **M**) Histograms showing the distribution of the expression of each transcript (Oct4 and miRNA145) in 1,094 hESC single-cells. Dash line indicates the gene mean copy number. Modified from [178].

As already mentioned, single stem cell analysis is favorable because it reduces the effect of the intrinsic heterogeneity of stem cells [176]. Microwell arrays provide a powerful tool for the single-cell analysis [177], but they are prevalently based on microscopy and image analyses in static conditions. We will here discuss methods that avoid cell-imaging analysis. The integration of microfluidic and qRT-PCR system allows the high throughput molecular analysis of single cells (Figure 9). White *et al.* [178] used this technique to separate single cells and monitor microRNA (miRNA) expression. These are important gene expression modulators involved in development and tumor formation and the ability of dissecting their expression from single cells open new possibility in the tumors treatments (for a review see [179]). Using the same principle, Zhong *et al.* [180] analyzed the expression of B2M, Nodal and Fzd4 genes of hESCs evidencing that gene expression data measured from a cell population is not a good representation of the expression levels in individual cells.

The association of microfluidics and microfabricated electrodes allows electric sorting and recovery of single live cells. This is particularly important to recover specific live cells from samples containing less than a few thousand cells. An example of relatively rare cells is adult stem cells. We analyzed, through a finite elements model and experiments, the sensitivity of different sensor topologies to the detection and the quantification of cells flowing in the test chamber [181–183]. This analysis suggests important parameters in the design of microsensors and presents a novel microfabrication technique for the development of 3D micropillars in flow chambers. Three different micropillar geometries with 50 μm height were compared. The work demonstrated that one single cell can be detected in a 450 μm wide chamber thanks to the employment of multiple interdigitated electrode pairs. Another method for the analysis of single cells is electrophysiological properties monitoring. Patch clamp chips allow performing measurements in a high throughput fashion, reviewed in [184]. It is interesting that companies such as Nanion Technologies GmbH, Munich; Cytocentrics AG, Ros- tock; Flyion GmbH, Tübingen; Essen Instruments (now Essen BioScience, Inc.), Cytion SA (Lausanne, acquired by Molecular Devices, LLC); Cellectricon AB (Mölndal); Sophion A/S (Copen- hagen); Fluxion LLC (San Francisco); Axon Instruments, now part of Molecular Devices, LLC (MDS) are all involved in the market of these devices. In fact, the technology for automated patch-clamp electrophysiology technology has been referred to as an “enabling technology” for ion channel drug discovery especially for screening drugs for cardiac ion channels safety.

Future perspectives on single cell analysis in association with microfluidic devices will be the spatial separation of molecules secreted from different cells once these molecules are detected electrically, in order to understand the activity-dependent molecular dynamics that occur in cells.

## 5. Conclusions/Outlook

Human stem cells and stem cells in general hold the potential to revolutionize nowadays medicine, leading to the development of novel therapeutic strategies and providing a reliable platform for performing drug-screening studies. Stem cells inside an organism reside in a complex microenvironment, formed by different inter-communicating compartments characterized by specific spatial and temporal parameters. The modulation of these complex signals is what determines cell behavior, and the control over such variables would allow fully unlocking the regenerative potential of stem cells. The tools described in this review represent noteworthy advances in the field of stem cell research thanks to their capacity of either controlling the cell microenvironment, measuring relevant physiological parameters and recording cell responses following defined stimulations. Advances in label-free technologies, described in this review, allow the analysis of cell behavior without modifying their physiological state and making them indispensable platforms in cell biology studies. Another field that would greatly benefit from the successful application of these technologies is that of novel drugs development. The extremely lengthy and costly processes of drug development for pharma industry would be revolutionized by the use of high throughput screening devices and platforms in which the sensing element is the human cell. The analysis of single cell through a new generation of cell chips is a challenging research because it opens new possibilities, among others, in tumors treatments studies, reducing the effect of the intrinsic heterogeneity of stem cells and cancer stem cells that are able to generate tumors through the stem cell processes of self-renewal and differentiation into multiple cell types. Another natural step in this direction would then lead to the effective establishment of the so-called personalized medicine. Being able to use our own stem cells as mimics for our organs and/or pathological conditions, each individual would potentially obtain *ad hoc* tailored therapeutic strategies to treat a specific disease, and study the effect of various drugs on its own target organs and systems.

## Figures and Tables

**Table 1.** Differential potential ranges of stem cells.

**Differentiation Potential**	**Number of Cell Types**	**Stem Cells**	**Differentiated Cells**
Totipotential	All	Zygote (fertilized egg), blastomere (ESCs)	All cell types
Multipotential	Many	Bone marrow cells	Skeletal muscle, cardiac muscle, liver cells, all blood cells
Oligopotential	Few	Myeloid or lymphoid precursor	Blood cells (Monocytes, macrophages, eosinophils, neutrophils, erythrocytes)
Nullipotential	None	Terminally differentiated cell e.g., Red blood cell	No cell division

**Table 2.** Effect of micropattern shape in cell behavior. Modified from [105].

**Cell types**	**Conditions**		**Cell fate**	**References**
	ECM micropattern	Biochemical cues		
Mesenchymal Stem Cells	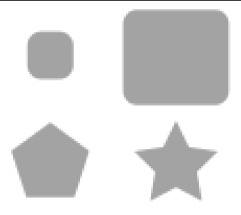	Adipogenic and osteogenic medium	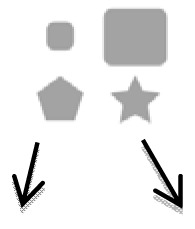 Adipocytes Osteoblasts	[106,107]
	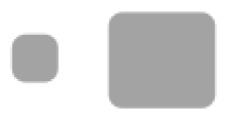	TGFβ	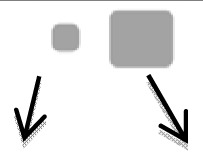 Chondrocytes Myocytes	[108]
Epidermal Stem Cells	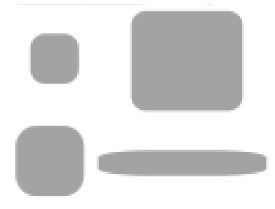	Growth factors	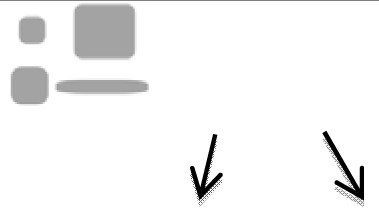 Epidermal cells Epidermal stem cells	[109]
Epithelial Cells	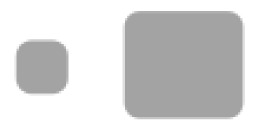	Matrix metalloproteinase 3 Low conc. TGFβ	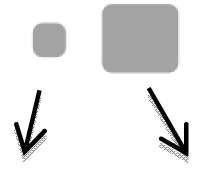 Epithelial cells Mesenchymal cells	[110,111]

**Table 3.** Comparison of methods for microgel fabrication.

**Method**	**Advantages**	**Disadvantages**
Micromolding	Controlled shape and size. Easy fabrication.	Batch process. Masks production. Uneven surface.
Photolithography	Controlled shape and size.	Batch process. Cell toxic photoinitiator. Cost for photolithograph masks.
Microfluidic	Homogeneous, continuous.	Non scalable. Microfluidic fabrication. Limited geometry due to pressure drop, PDMS affinity for small hydrophobic molecules.
Emulsification	Easily scalable.	Limited to spherical shapes.

**Table 4.** Detection methods used in different electronic cell microchips.

**Detection Method**	**Cell Information**	**Reference**
Impedance (EICS)	Cell shape (normal, apoptosis, necrosis, swelling, lysis, size), motility (migration, tumor cell infiltration, invasion), differentiation, spreading, adherence, epithelial membrane integrity and polarity.	[185–190]

Amperometric (MEA)	Cell secretion (metabolites, exocytosis).	[191]

Capacitive (MEA)	Membrane structure and activity.	[192]

Potentiometric (LAPS)	Extracellular potentials, cell metabolism analysis.	[159,160]

Patch-clamp array	Ion channels activity from single cells.	[193]

FET	Extracellular/intracellular current, electric signals, cell-cell communication.	[144]

Refraction index (SPR)	Cell adhesion, morphology, motility.	[167]

Piezoelectric effect (QCM)	Cell attachment, proliferation, shape, substrate interaction.	[169]
